# Perceptual no-reference image quality assessment with meta-learning by graph representation learning and multi-scale feature fusion

**DOI:** 10.1371/journal.pone.0351549

**Published:** 2026-06-18

**Authors:** Yanru Jia, Longsheng Wei

**Affiliations:** 1 School of Computer Science and Artificial Intelligence, Xinyang College, Xinyang, Henan, China; 2 School of Artificial Intelligence and Automation, China University of Geosciences, Wuhan, China; Chitkara University Institute of Engineering and Technology, INDIA

## Abstract

No-reference image quality assessment (NR-IQA) aims to predict perceptual quality in alignment with the human visual system (HVS), yet existing methods face challenges in capturing long-range dependencies across distortion types and levels while preserving content fidelity during preprocessing. This paper presents a perceptually-driven NR-IQA framework that integrates meta-learning, graph representation learning, and multi-scale feature fusion to address these limitations. First, a meta-learning paradigm is employed to pre-train a self-calibrated convolutional backbone, which adaptively models spatial and channel-wise dependencies across scales, thereby enhancing the extraction of distortion-aware features while mitigating information loss caused by fixed-input preprocessing. Second, a graph representation learning module is introduced to explicitly encode the hierarchical relationships among distortion types, distortion levels, and image content. Nodes in the graph correspond to distorted images, while edges capture inter-sample similarities; these are jointly optimized via a graph convolutional network under dual supervision from a triplet-based distortion-type discriminator and a probabilistic distortion-level regressor that accounts for content-induced uncertainty. Extensive experiments on four benchmark datasets demonstrate that our method achieves better performance, with average SROCC and PLCC improvements of 3.6–36.6% over hand-crafted feature-based methods and consistent gains over deep learning-based approaches. Ablation studies and visualizations confirm that the proposed components collectively yield a more discriminative and generalizable distortion representation, closely mirroring human perceptual judgments.

## Introduction

With the development and popularization of electronic information technology and intelligent devices, multimedia digital images have been widely used as carriers of information transmission in medical and health care, education and life, weather forecasting, etc. However, pictures may suffer from varying degrees of distortion throughout the transmission, compression, and storage processes, resulting in image quality reduction. Therefore, image quality assessment (IQA) [1] has always been an important research topic in the field of computer vision, and have wide applications in image retrieval image compression, image fusion, virtual reality and other fields.

Within the context of IQA, no-reference IQA (NR-IQA) has received widespread attention because reference is often not available in lots of real-world application scenarios. At the same time, the robustness of learning-based NR-IQA metrics can be attributed to the powerful fitting capability of deep convolutional neural networks (DCNN) [2]. They represent degradation by automatically capturing depth features and have been widely used for NR-IQA tasks. However, DCNN uses end-to-end training to establish complex relationships between image distortion and model parameters, and it is easy to ignore the detailed information present in distorted images, so the DCNN-based approaches also have the following problems.

On the one hand, in a distorted image obtained in reality, there will be not only global uniform distortion (e.g., low exposure, out of focus) but also non-uniform distortion in local areas (e.g., target movement, ghosting). Ignoring the link between local information and the global image increases the error in IQA [3]. Traditional CNN generally perform convolution operations in small regions (e.g., 3 × 3), focusing mostly on distorted information of local relationships, and are unable to acquire globally relevant features. On the other hand, an unavoidable problem with deep learning solutions is the fixed-scale input required for end-to-end models [4]. Although many preprocessing approaches have been proposed to address this issue, inappropriate processing reduces the consistency between images and their true quality scores. The cropped images contain different level and type of degradation from the original image, while rotation of the image will introduce unrealistic colors that will have negative impact on the image quality. And rescaling distorts the image subject, thus affecting the semantic content so that the true quality score no longer applies to the pre-processed image. Therefore, using these pre-processed images to train the model may lead to biased predictions.

In cases where labeled data is difficult to obtain, a good distortion representation can help train the target task. The type of distortion an important factor affecting the quality of image perception [5]. Although there are studies adding type classification tasks that can assist IQA, these methods are unable to distinguish the level of distortion and ignore the intrinsic distributional properties between distortion levels. Therefore, existing distortion representation methods remain limitations, whereas graph convolutional network (GCN) [6,7] can use the concept of a graph to describe the connections between each individual. The input of GCN consists of two parts, node and edge information, with nodes representing individuals and edges quantifying the relationships between individuals.

To design a model more in line with human perception, we propose an approach through GCN and multi-scale features, using representational learning to evaluate image quality. More specifically, our model is based on a meta-learning framework for pretraining model parameters to further improve the generalization of distorted data. To enhance the local-global connection, an adaptive module for local and global fusion is designed. The correlation between larger perceptual field space and channels is constructed adaptively for each spatial location, and long-range features are exploited to guide the original features for feature variation. Finally, the distortion-related factors affecting perceptual quality are then modeled using GCN to obtain a more discriminative distortion hierarchical representation.

Our work has three main contributions:

1. Design an adaptive multi-scale feature fusion module to adaptively model the global dependency relationship between spatial and channel dimensions, organically combining local detail features with global structural information, significantly improving the model’s perception accuracy and feature expression ability for distorted regions, and making the extracted features more in line with HVS.
2. Adopting a meta learning strategy based on double-layer optimization, pre training is conducted on a synthetic distortion dataset to learn cross task transferable distortion common priors and general initialization parameters, enabling the model to quickly adapt to new distortion types and evaluation tasks, and to obtain shared quality perception knowledge through meta learning.
3. Construct a graph representation learning module that uses distorted images as graph nodes and similarity between samples as graph edges. Utilize graph convolutional networks to explicitly model the hierarchical relationship between distortion types, levels, and image content, enabling the model to learn more discriminative distortion representations.

## Related work

### NR-IQA methods

The NR-IQA methods can be divided into distortion-specific [8] and general algorithms [9]. Distortion-specific metrics quantify image quality by describing known levels of distortion. Due to the known distortion type characteristics, these metrics provide significant evaluation performance. However, in practice, the nature of image distortion is often unknown, limiting the range of these metrics [10]. Therefore, general NR-IQA metrics have become the focus of researchers’ attention in recent years.

Traditional generic NR-IQA models are generally based on hand-crafted functions and are classified into two types: natural scene statistics (NSS)-based [11] and learning-based [12]. The statistical features of natural photographs fluctuate depending on the degree of distortion, according to NSS-based measures. Mittal *et al.*[13] presented BRISQUE and achieved good performance by extracting NSS properties in the spatial domain. Zhang *et al.* [14] proposed the unsupervised learning NR-IQA model ILNIQE by introducing structural features, frequency features and color features. Apart from the measures mentioned above, learning-based metrics are also becoming more and more popular. Inspired by the success of machine learning in many computer vision tasks [15], the traditional distance-based mass pooling approach was replaced by automatic learning with the introduction of dictionary learning methods [16].

Recently, the general NR-IQA algorithm based on deep learning has attracted considerable attention thanks to its efficient and adaptive ability to extract distorted perceptual feature [17]. To compensate for the lack of data, Kim *et al.* [18] proposed BIECON, which uses the local quality map obtained by the FR-IQA algorithm as an intermediate result to train the model. Bosse *et al.* [19] proposed WaDIQaM, which pools image local block quality into global image quality and achieves good results on synthetic distortion databases. Liu *et al.* [20] proposed RankIQA, an NR-IQA method based on ranking learning, which uses Siamese neural networks to learn the ranking of two images sampled from the same distortion. However, RankIQA ignores to model distortion types. Ma *et al.* [21] designed dipIQ, a fully connected neural network model based on weight sharing. A large number of training samples were generated by labeling quality classes instead of quality scores, and RankNet was used to learn the rank of images. CaHDC [22] used cascaded architecture to export features at different scales. Due to the diversity of distortion and content, these methods can still observe significant performance degradation in real distortion applications.

More methods for authentic distortion have emerged since synthetic distortion-based IQA methods are often not effectively used in real distortion. Zhang *et al.* [5] attempted to directly bind synthetic and real feature sets bilinearly in hopes of improving performance when processing both types of skewed data. However, in order to manage both artificial and genuine biases, this method needs two pre-trained networks. Additionally, the bilinear pooling approach has a significant computational cost. Considering the impact of image content on human visual system (HVS), Su *et al.* [23] proposed an adaptive hypernetwork to cope with real distortion, which divides the IQA process into three steps: content understanding, perceptual rule building, and quality prediction. Compared with CNNs, Transformer is able to construct non-local feature representations, so You *et al.* [24] first applied Transformer to the field of IQA, revealing that non-local information plays a crucial role in IQA. Zhu *et al.* [25] proposed a saliency-guided Transformer network that adds the gradient of traditional feature maps to complement local information, achieving excellent evaluation performance on real distortions. However, the Transformer -based methods only consider the spatial feature distribution but ignore the effect of inter-channel information.

Finally, while CNN-based NR-IQA approaches often depict geometric connections poorly and describe distortion kinds or degrees as simple ranking models, deep learning-based NR-IQA methods outperform traditional hand-designed feature-based NR-IQA methods. In addition, CNNs focus on local features and it is difficult to effectively aggregate non-local features to perceive image quality.

### Graph representation learning

The CNN-based approaches focus on local features, while graph neural networks, which aim at non-local quality perception, are more useful for modeling the relationships between different regions. As an extension of convolutional networks, GCN use the concept of graphs to describe the connections between each individual. Sun *et al.* [26] introduced GCN to construct distortion graph representations to distinguish different distortion types by comparing different distortion graphs, while learning the relationship between distortion levels to predict image quality. However, this model is not specifically designed to extract global features. Huong *et al.* [27] applied GCN to solve the quality evaluation problem of virtual reality (VR) images. Pan *et al.* [28] proposed a hybrid network for reference-free video quality evaluation to explore the relationship between image frames in videos by GCN. Jia *et al.* [29] designed a super-pixel-based GNN approach to capture nonlocal features and explore nonlocal interactions in quality prediction.

Inspired by the above approaches, in order to construct an effective representation of image distortion, we model the relationship between distorted images using GCN, while modeling the global dependencies of local features and the hierarchical relationship of distortion information.

### Multi-scale feature fusion

Multi scale feature fusion integrates multi-level features from local details to global structure of images, compensating for the deficiency of insufficient expression of single scale features, enabling the model to comprehensively perceive image degradation of different scales and types. It not only preserves high-frequency distortion clues sensitive to the human eye, but also takes into account overall semantic integrity and structural consistency, effectively alleviating the problem of insufficient representation caused by missing reference information in NR-IQA tasks, and enhancing the robustness and cross dataset generalization ability of the model under mixed distortion. Zhang *et al.* [30] propose a multilevel Chebyshev stack structure. The generated structure not only benefits in exploring the local and global geometric features along horizontal, vertical, left, and right-diagonal directions but also maintains the view correlation consistent in each angular direction. Liu *et al.* [31] employs 3D convolution for the joint extraction of spatial and angular information, leveraging their interdependencies for more effective reconstruction.multi-representation enhancement block enhances features by learning pixel differences across multiple directions in diverse representations, effectively capturing intricate details and complex correlations. Mao *et al.* [32] proposed a sparse to dense multi-scale progressive fusion model to address the problem of rough fusion of multi-scale views and ineffective reconstruction of occluded areas.

Taking inspiration from the above methods, we design an adaptive multi-scale feature fusion module that adaptively models the global dependency relationship between spatial and channel dimensions, organically combining local detail features with global structural information to make the extracted features more in line with HVS.

## Our approach

**Fig 1:**
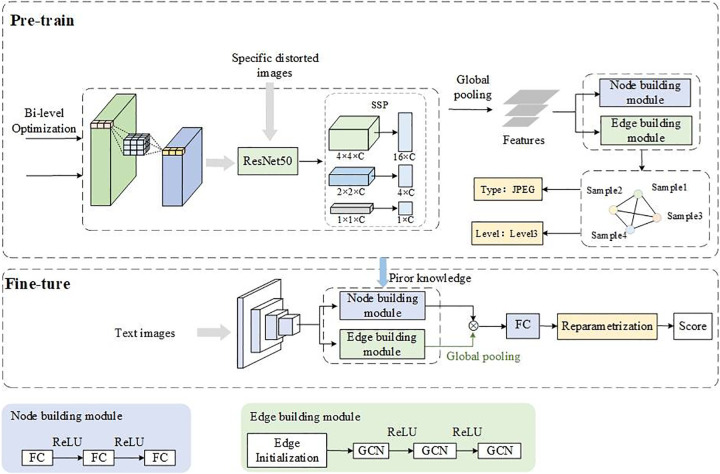
An overall framework of our model.

In this paper, we propose an approach to address the problems of traditional convolutional networks and construct the distortion representation that is more in line with the process of image evaluation by the human eyes. The specific framework is shown in Fig 1, including two parts: pre-training and fine-tuning.

In the pre-training phase, in order to enhance generalization capability in unknown distortion scenarios, we introduce a meta-learning framework to obtain the meta-model with shared quality prior knowledge and use an optimization-based approach [33] to learn the initialization parameters and optimization rules general to the model network from the synthetic datasets, adapting to the unknown distortion through bi-level gradient optimization. Consequently, various distortion tasks for particular distorted images are first gathered to create a meta-training set, and each job is divided into a query set and a support set. Next, the stochastic gradient descent approach is applied using Adam to compute the gradients of the model parameters on the support set and update it, and lastly, the query set is used to verify that the modified model parameters can be executed successfully. The meta-learning technique can increase prediction accuracy and enhance the capacity of various skewed datasets to generalize. Following the meta-model, an adaptive fusion of local and global features module is added to extract features, expanding the field of perception of the convolution operation, building global spatial and inter-channel dependencies, and mining more rich distortion features. After training the meta-model containing specific distortion knowledge, the Spatial Pyramid Pooling module is added to receive images of arbitrary size. The GCN is then used to obtain the graph representation of the specific distortion type, and the two discriminatory modules of distortion type and distortion level are used to optimize the distortion graph representation and obtain prior knowledge for constructing the distortion graph representation. Finally, when fine-tuning on the target NR-IQA tasks, a good representation of distortion-related factors can be constructed quickly without optimization.

### Meta-model incorporating multi-scale features

**Fig 2:**
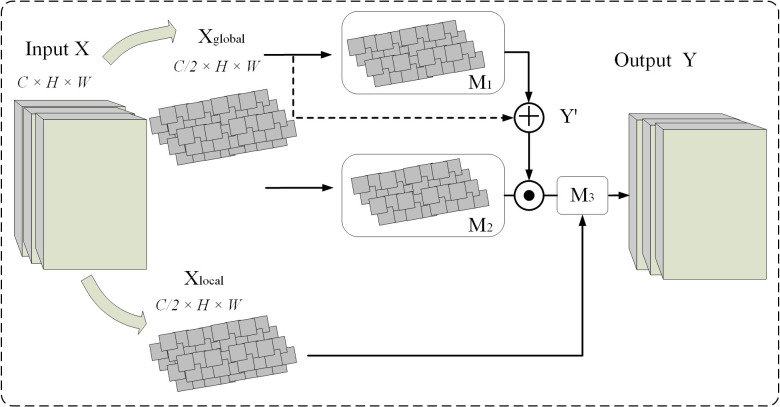
Adaptive fusion multi-scale features module.

Human vision in perceiving distorted images not only perceives the semantic information of the image as a whole from a global perspective but is also able to perceive the local details of interest in the image. However, there is a strong generalization bias, i.e., localization, in convolutional neural networks (CNNs), and therefore it is difficult to capture the dependencies of local pixels and non-local features. To address the problem that CNNs cannot increase the receptive field of neurons to model non-local features, inspired by SCNet [34] networks, This paper considerably increases the receptive field of each convolutional layer through internal communication to improve its representational learning potential. First, the DCNN model is trained to accommodate unknown distortions by bi-level gradient optimization in a meta-learning framework. Meanwhile, during feature extraction, the convolutional block is divided into multiple parts, and the transformation of one convolutional block is used to calibrate the feature changes in another part of the block, thus effectively expanding the receptive field at each spatial location and adaptively constructing global spatial and inter-channel dependencies. The ability to enhance local feature representation with global information allows more discriminative distorted features to be captured, resulting in more accurate image quality scores predicted by the model. The specific process is shown in Fig 2, where different operations are performed through the three convolutional layers to obtain local and global feature information.

For a set of feature layers of the given shape (*C*,*H*,*W*), they are divided into 3 convolutional blocks $\left\{M_{1},M_{2},M_{3}\right\}$. The input *X* is then divided into $\left\{(X_{local},X_{global})\in R^{C/2\times H\times W}\right\}$ using a channel split operation, and different operation paths are performed using $X_{local}$, and $X_{global}$, respectively. In the first path, $\left\{M_{1},M_{2}\right\}$ is used to perform a calibration operation on $X_{global}$. Given an input $X_{global}$, an average pooling of size   R×R  and step size *R*  is used to expand the perceptual field:


$$\begin{array}{c} P_{1}=AVGPOOL_{R}(X_{global}) \end{array}$$ (1)

The feature transformation is then applied to *P*_1_ by *M*_1_:


$$\begin{array}{c} X_{global}^{\prime }=UP(P_{1}\times M_{1}) \end{array}$$ (2)

where UP(·) is the bilinear interpolation operator, and after the feature change is performed, a calibration operation is added to enhance the mapping of the feature map to the distorted regions of the original image. Channel enhancement is performed by  *M*_2_  for  $X_{global}$ :


$$\begin{array}{c} Y^{\prime }=(X_{global}\times M_{2})\cdot \alpha (X_{global}+X_{global}^{\prime }) \end{array}$$ (3)

where   α  is the activation function and  ·  is the dot product of the features. Finally, using   $X_{global}^{\prime }$  as a residual factor, the distortion information is effectively captured, and the global distortion information captured is fused with the original feature   $X_{local}$  by *M*_3_ to obtain the output vector *Y*:


$$\begin{array}{c} Y=(Y^{\prime }⊕X_{local})\times M_{3} \end{array}$$ (4)

The advantage of adaptively fusing multi-scale meta-model is that each spatial location not only allows global contextual information to be considered adaptively as a potential spatial embedding in the original space to guide its change, but also allows dependencies between channels to be modelled, effectively building links between local information and global context and enhancing the ability of convolutional neural networks to model global relationships.

### Graph representation learning

Representation Learning is a fundamental part of deep learning. Deep learning methods’ performance is determined by the quality of the data and effective representation, so it is of great importance to represent the important factors affecting HVS in a more concise and superior way. Most present methods treat various distortion types as a flat model and do not model the link between distortion type and degree, nor do they take into account the impact of image content on perceptual quality. Previous research has found that image quality scores with the same distortion type and intensity follow a Gaussian distribution [28]. Moreover, human eyes see the relationship between distortion type and level as a hierarchical model when evaluating distorted pictures. Based on this, this study presents a hierarchical model that illustrates the relationship between picture distortion type and level, where each form of distortion is represented by a graph structure. To avoid arbitrary changes in the input size leading to changes in the quality of the original image, we introduce the SPP module into the meta-model structure to construct the NR-IQA backbone. The SPP operation is performed on the last convolutional layer of the meta-model, such that a fixed image size is ensured for the output, regardless of the input image size.

In this paper, graph representations of distorted images are constructed using GCN, where the node represents an image and the edge represents the relationship between images. The graph representation for distortion type  *k*  can be defined as  $G_{k}=(V_{k},E_{k})$, *V*  for nodes and  *E*  for edges. First, we extract  *N*  specific distorted images into the model to obtain the corresponding feature set  $F_{k}=\left\{f_{i}|i=1,2,...,n\right\}$ . The feature vector  $f_{i}$  of each image is used to initialize the nodes, and the similarity between each image is used to initialize the edges. The distorted images are represented by the node and edge information to represent the graph structure of the specific distorted image, as follows.

#### Node building for graph representation.

To contain more distortion-related information for each node between different distortion types, this paper uses a node building module consisting of fully connected (FC) layers to optimize, as shown in Fig 1, the optimization process is as follows:


$$\begin{array}{c} V_{k}=\left\{V_{k,i}|v_{k,i}=F_{N}(f_{k,i};\theta ),v_{k,i}\in R^{C},i=1,2,...,N\right\} \end{array}$$ (5)

where  $v_{k,i}$  denotes the node generated by the  *i*  -th sample and  θ  denotes the network parameters of the node building module.

#### Edge building for graph representation.

To obtain more contrasting relationships between nodes and thus distinguish different levels of distortion between the same distorted images, this paper initializes the value of the edge feature  $E_{k}^{0}$  by the dot product between nodes, denoted as  $E_{k,i,j}^{0}\in R^{C}$ ,with  *i*,*j*  denoting the relationship between the  *i*  -th and the  *j*  -th sample. After initializing the edge features, the internal structure of the graph representation is further optimized by inputting the edge building module composed of GCN. To normalize the output, the activation function uses ReLu, as shown in Fig 1. The initialized edge features  $E_{k}^{0}$  and the corresponding adjacency matrix  $A_{E_{K}}\in R^{N^{2}\times N^{2}}$  is used as input and the values of the edge features obtained after optimization by the  *L*-layer GCN can be calculated as:


$$\begin{array}{c} E_{k}^{l+1}=\sigma ({\hat{A}}_{E_{k}}E_{k}^{l}W_{E}^{l}) \end{array}$$ (6)

where, σ denotes the ReLu operation, $D=diag(\sum_{n=1}^{N^{2}}(A_{E_{k}}+I{)}_{p})$, ${\hat{A}}_{E_{k}}=DA_{E_{k}}D$, and $E_{k}^{l}$ denote the output of the *l*-th layer of the GCN, $W^{l}$ is the weight of matrix. Finally, the set of edge features represented by the distortion graph can be defined as:


$$\begin{array}{c} E_{k}=\left\{e_{k,i,j}|e_{k,i,j}\in R^{C_{E}},i,j=1,2,...,N\right\} \end{array}$$ (7)

where $C_{E}$ denotes the dimension of the edge $E_{k}$.

The whole procedure of the proposed model is summarized in Algorithm 1.

**Algorithm 1**

**Input:** Input dimension: $D_{in}$, Domain embedding size: $S_{d}$, Edge embedding size:$S_{e}$

**Output:** Domain embedding: $E_{d}$, Processed edge embedding: $E_{e}^{\prime }$, Level prediction: Y, Updated domain embedding:$E_{k}$

1:  Initialize variables

2:  Initialize pyramid pooling layer

3:  Initialization point processing GCN-V and edge processing GCN-E

4:  Applying GCN-V to process node features and obtain domain embeddings$E_{d}$

5:  Apply GCN-E to process edge features and obtain edge embeddings$E_{e}$

6:  **if** pre-train is true than **then**

7:  Reshaping $E_{e}$ to fit the size of $E_{d}$. Output as $E_{e}^{\prime }$

8:   Double-layer gradient optimization

9:  **else**

10:  Reshaping $E_{e}$ and filtering it through the identity matrix to obtain $E_{e}^{\prime }$

11: **end if**

12: Calculate level prediction Y

13: Update domain embedding $E_{k}$

#### Optimization of graph representation.

**Fig 3:**
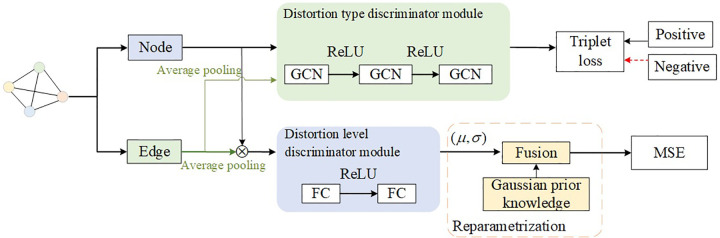
Optimization of graph representation.

After the distorted image is represented as a graph structure, this paper continues to optimize it in the following two ways. a) In order to learn the distortion information of different distortion types and the comparison relationship between various distortions, so as to achieve better generalization performance, we propose a distortion type discrimination module; b) Considering the influence of image content, in order to simulate the distribution of subjective quality scores under the same distortion type and degree, a distortion level discriminator module is proposed in our method. The specific structure is shown in Fig 3.

a) Distortion type discrimination module

To obtain typical features of each distorted graph representation and thus distinguish between the types of distortion represented by different graph structures, with the following implementation method, we continue to use numerous layers of GCN to collect global information from the set of node features and inter-sample associations from the set of edge features:


$$\begin{array}{c} V_{k}^{l+1}=\sigma ({\hat{A}}_{V_{k}}V_{k}^{l}W_{T}^{l}) \end{array}$$ (8)

where an average pooling transformation of  $E_{k}$  yields the adjacency matrix  $A_{V_{k}}\in R^{N\times N}$  of node  $V_{k}$ . The output of the distortion type discriminator is a vector of dimension  $C_{v}$  denoted  $y_{code}$ . In order to aggregate distortion graph representations with the same distortion while separating different distortion types, we use triplet loss to learn comparative representations of different distortion types. In detail, three forward propagations are performed to obtain a ternary with three sets of inputs. As shown in Fig 3, the graph representation output by the distortion type discriminator is used as the baseline sample and is denoted as Anchor, while samples of the same type as Anchor are denoted as Positive and samples of the different type from Anchor are denoted as Negative. By inputting the three sets of samples, the loss function  $L_{type}$ can be calculated as.


$$\begin{array}{c} L_{type}=\max\left\{d(y_{code},y_{code}^{p})-d(y_{code},y_{code}^{n})+margin,0\right\} \end{array}$$ (9)

where  *d*  denotes the Euclidean distance,  $y_{code}^{p}$  denotes the Positive sample, while  $y_{code}^{n}$  denotes the Negative sample and  *margin*  denotes the threshold that separates the Positive from the Negative in the comparison. Here, $L_{type}$  not only learns the finer differences between distortion types, but also the contrasting relationships between distortion types to avoid overfitting the network to the distortion types in the training set.

b) Distortion level discrimination module

To forecast the level of distortion while accounting for the uncertainty generated by the image content, the distortion level discrimination module is designed in this paper. Sample scores at the same level are distributed using the Gaussian distribution around the mean score when distorted images have the same distortion kind and amount. Perceived image quality changes depending on content. Based on this, this paper performs predictions by sampling randomly from a Gaussian prior distribution $N(\mu ,\sigma^{2})$ , where  μ and σ are the mean and standard deviation of the predictions generated by the distortion level discriminator module. The Reparametrization Trick is used to make sure the network is trained all the way through because this process is not simple. In particular, ϵ is sampled from a basic Gaussian distribution *N*(0,1) and then translated to an arbitrary Gaussian distribution using the produced hyperparameters *epsilon* :


$$\begin{array}{c} y_{i}=\mu_{i}+\sigma_{i}\varepsilon ,\varepsilon \in N(0,1) \end{array}$$ (10)

Since the prediction of distortion levels is achieved not only by analyzing the information contained in the node features but also by comparing nodes with each other, both node and edge information need to be input. Specifically, the node features $V_{k}$ are first fed into the distortion level discriminator module, and then the edge features $E_{k}$ are input through averaging pooling and expressed as $E_{k}^{\prime }=[\sum_{j}e_{k,i,j}]$/N . The current node’s data is combined with that of the other neighboring nodes in these two branches. Lastly, the distortion level discriminator module is trained using the Mean Square Error (MSE) loss function:


$$\begin{array}{c} \begin{array}{c} L_{level}=\sum_{i}|y_{i}-y_{i}^{\prime }{|}^{2} \end{array} \end{array}$$ (11)

where  $y_{i}^{\prime }$  represents the true degree of distortion. The loss function of the overall model is obtained by weighting the above loss function by the hyperparameter  λ  as:


$$\begin{array}{c} L=L_{type}+\lambda L_{level} \end{array}$$ (12)

### Fine-tuning of the graph representation

Due to the improved feature representation capabilities of the graph structure, the method in this paper is able to perform the IQA task better. Both the node building module and the edge building module are used to regress IQA scores while fine-tuning the target datasets, eliminating the need for the graph representation optimization component, as shown in Fig 1. Specifically, the outputs of the node building module and edge building module, which are learned with rich prior knowledge, are stitched together and subsequently fed into the FC layer to predict the final perceptual quality score. The loss function for the training model during fine-tuning uses the MSE, defined as:


$$\begin{array}{c} L_{f}=\frac{1}{N_{f}}\sum_{i=1}^{N_{f}}|y_{i}-y_{i}^{\prime }{|}^{2} \end{array}$$ (13)

where  $N_{f}$ denotes the mini-batch size. When fine-tuning the pre-trained graph building model on the authentic datasets, the quality assessment of the authentic images is achieved based on the Gaussian prior distribution, thus improving the prediction accuracy for unknown distortion types.

## Experimental analysis

The experimental setup of the proposed approach, as well as the experimental findings on publicly accessible quality evaluation datasets, are described in this part. The experimental setup includes model training, dataset selection, and evaluation metrics. The experimental procedure includes performance evaluation of the overall dataset, performance evaluation of single distorted dataset, and the ablation experimental study.

### Implementation details

The training approach for the suggested technique in this study is divided into two stages: (1) during the pre-training phase, the meta-model network fusing multi-scale features and constructing network of graph representation are trained on the synthetic dataset; (2) during the fine-tuning phase, the overall network is trained on the target datasets and the parameters are fine-tuned.

The pre-trained meta-model is acquired using the meta-learning framework and utilized to build the feature extraction network during the pre-training phase. The Kadid-10k dataset is then used to train the whole model network [35]. The hyperparameter λ of the loss function is set to 0.25 and the margin of the triple loss $L_{type}$ is set to 0.1. The network parameters are iterated 40,000 times using optimization method, with a mini-batch size of 64 and a learning rate of 1e−7 . The dimension size $C_{v}$ of the node building network is set to 256 and the size of $C_{E}$ in the edge building network is set to 64.

For the target datasets during fine-tuning, two datasets with authentic distortion [36,37] and two datasets with synthetic distortion [38,39])were selected in this paper. For fine-tuning, the datasets were split into training and test sets. Specifically, 80% of the photos from the legitimate datasets KonIQ-10k and LIVEC were chosen at random as the training set, while 20% were utilized as the test set. Divide the dataset into a training set and a testing set based on the reference image with a ratio of 8:2 to make the image content independent in the training and test sets. All results are obtained by training and testing 10 random segmentation operations on the specific target dataset and the average results are reported. The target IQA task is fine-tuned 20 times in this study using the Adam optimization method with a mini-batch size of 32 and a learning rate of 5e−6.

### Performance evaluation on the overall datasets

Eleven exemplary IQA methods were chosen for experimental comparison in order to assess the prediction accuracy of the approaches in this research, including the manual feature extraction-based methods [13,14,16], the deep learning-based synthetic distortion IQA methods [18,19,22] and the deep learning-based authentic distortion IQA methods [5,23,40–50], and the experimental results are shown in Tables 1 and 2, with the best results highlighted in bold.

**Table 1:** SROCC results for different NR-IQA methods.

SROCC	LIVEC	KonIQ-10k	CSIQ	LIVE	Average
BRISQUE [13]	0.606	0.648	0.746	0.938	0.735
ILNIQE [14]	0.432	0.507	0.806	0.901	0.662
HOSA [16]	0.645	0.670	0.751	0.945	0.753
WaDIQaM [19]	0.671	0.745	0.955	0.954	0.831
CaHDC [22]	0.734	0.819	0.903	0.965	0.855
SFA [40]	0.810	0.839	0.796	0.884	0.832
DBCNN [5]	0.852	0.872	0.946	0.967	0.909
HyperIQA [23]	0.859	0.905	0.923	0.962	0.912
MetaIQ [41]	0.802	0.850	–	–	0.826
MetaIQA+ [42]	0.852	0.909	–	–	0.881
EDIIQA [43]	0.788	0.901	0.952	0.972	0.903
VISOR [44]	0.846	0.896	0.961	0.973	0.919
TempIQA [45]	0.870	0.903	0.950	0.976	0.925
CLIP-IQA [50]	0.656	0.695	0.748	0.626	0.681
QualiCLIP [47]	0.725	0.817	0.804	0.898	0.811
HiRQA [48]	0.692	0.802	0.862	0.902	0.814
AKD-IQA [46]	0.850	0.896	0.952	0.968	0.917
FsPN [49]	0.860	**0.918**	0.955	0.979	0.928
**Ours**	**0.881**	0.910	**0.962**	0.979	0.933

**Table 2:** PLCC results for different NR-IQA methods.

PLCC	LIVEC	KonIQ-10k	CSIQ	LIVE	Average
BRISQUE [13]	0.621	0.678	0.829	0.935	0.766
ILNIQE [14]	0.508	0.523	0.808	0.865	0.676
HOSA [16]	0.678	0.714	0.824	0.947	0.791
WaDIQaM [19]	0.680	0.798	**0.973**	0.963	0.854
CaHDC [22]	0.738	0.834	0.914	0.964	0.863
SFA [40]	0.834	0.872	0.818	0.895	0.855
DBCNN [5]	0.865	0.881	0.959	0.971	0.919
HyperIQA [23]	0.882	0.922	0.943	0.966	0.928
MetaIQ [41]	0.835	0.877	–	–	0.856
MetaIQA+ [42]	0.852	0.922	–	–	0.887
EDIIQA [43]	0.781	**0.929**	0.956	0.979	0.911
VISOR [44]	0.866	0.910	0.967	0.978	0.930
TempIQA [45]	0.886	0.920	0.960	0.977	0.936
CLIP-IQA [50]	0.670	0.730	0.805	0.651	0.714
QualiCLIP [47]	0.802	0.838	0.842	0.885	0.841
HiRQA [48]	0.744	0.812	0.874	0.890	0.830
AKD-IQA [46]	0.881	0.904	0.959	0.968	0.928
FsPN [49]	0.884	0.918	0.955	0.979	0.934
**Ours**	**0.889**	0.926	**0.973**	**0.982**	**0.943**

From Tables 1 and 2, it can be observed that the method proposed in this paper achieves SROCC results of 0.881, 0.910, 0.962, 0.979 and PLCC results of 0.889, 0.926, 0.973, 0.982 on the LIVEC, Koniq-10k, CSIQ and LIVE datasets, respectively. Our model achieved optimal results on average for all four datasets. For the Koniq-10k and LIVE datasets, On SROCC and PLCC, our technique had the highest prediction accuracy. The experimental data are then examined from three angles:

(1) Compared with traditional methods of manual feature extraction, our method largely outperforms such methods on all datasets. For the SROCC results, our method outperforms the more accurate HOSA method by about 36.59% on LIVEC, 35.82% on Koniq-10k, 28.10% on CSIQ, and 3.60% on LIVE. For the PLCC results, the method in this paper exceeds the next best HOSA method by about 31.12% on LIVEC, about 29.69% on Koniq-10k, about 18.08% on CSIQ, and about 3.70% on LIVE. From the experimental results, it is clear that relying on graph convolutional networks to construct distortion representation can extract richer distortion-related information than methods based on manual feature extraction, thus largely improving the prediction accuracy of the networks.
(2) Compared to deep learning-based synthetic IQA methods, our method outperforms such methods on both authentic datasets. For the SROCC and PLCC results, our method outperforms the more accurately predicted CaHDC by about 20.03% and 20.46% on LIVEC, by about 11.11% and 11.03% on Koniq-10k, by about 6.53% and 6.46% on CSIQ, and by about 1.45% and 1.87% on LIVE. It can be seen that although our method does not use real distorted data in the pre-training phase, it can perform well on authentic datasets. This suggests that the pre-trained model has the potential to perform the IQA task and can transfer the learned distortion experience to other distortion domains.
(3) Compared with authentic distortion IQA methods based on deep learning, our method achieves the highest prediction accuracy on all four datasets for the SROCC results. For the PLCC results, our method achieves the highest prediction accuracy on LIVEC, CSIQ and LIVE. On Koniq-10k, the EDIIQA approaches have better prediction accuracy than our approach. The main reason is that the bilinear pooling strategy of EDIIQA can cope with both synthetic and authentic distortions, whereas our method only uses a smaller synthetic dataset during the pre-training process.

Analysis of the combined experimental data leads to the conclusion that the distortion representation method used in this paper achieves better prediction accuracy than methods based on manual feature extraction. Compared with deep learning-based methods, we achieve multi-scale features fusion through augmented convolution and further aggregation of global sample information through GCN, which significantly improves prediction accuracy and generalization performance and is more consistent with human perception.

### Performance evaluation on single distortion datasets

Two datasets with synthetic distortion, were chosen to carry out single distortion type testing and to compare with other methods in order to validate the prediction accuracy of the approach in this study under different distortion types. The experiments include five distortion types in LIVE and six distortion types in CSIQ. The results of the SROCC on the LIVE dataset are shown in Table 3. On the LIVE dataset, our technique is seen to provide the best prediction accuracy for all four categories of distortion. For the distortion types JPEG, JP2K and GB, our method can effectively learn the degree of quality degradation for these distortion types as the dataset containing such rich distortion types when pre-training the model for constructing distortion graph representation. For the distortion types WN and FF, although the dataset of the pre-trained model does not contain such distortions, our method achieves SROCC results of 0.980 and 0.960. Our proposed meta-model with the adaptive fusion of multi-scale features can effectively capture distortion information and retain more image distortion information by fusing local area features with global features, with good generalization performance.

**Table 3:** SROCC results for different distortion types of images on the LIVE dataset.

SROCC	JPEG	JP2K	WN	GB	FF
BRISQUE [13]	0.965	0.929	0.982	0.964	0.828
ILNIQE [14]	0.941	0.894	0.981	0.915	0.833
HOSA [16]	0.954	0.935	0.975	0.954	0.954
WaDIQaM [19]	0.953	0.942	**0.982**	0.938	0.923
CaHDC [22]	0.963	0.948	0.977	0.953	0.907
DBCNN [5]	0.972	0.955	0.980	0.935	0.930
HyperIQA [23]	0.961	0.949	0.982	0.926	0.934
CLIP-IQA [50]	0.921	0.932	0.980	0.947	0.928
**Ours**	**0.975**	**0.968**	0.980	**0.975**	**0.960**

The results of SROCC on CSIQ are shown in Table 4. As can be shown, our technique obtains the highest forecast accuracy for the three CSIQ distortion categories, and achieves more satisfactory results in the evaluation of the remaining distortion types. For distortion type WN, the ability to characterize distortion features is weakened by the inability to effectively construct a link between local and global distortion features. Even in this case, our method still has better result compared to other methods.

**Table 4:** SROCC results for different distortion types of images on the CSIQ dataset.

SROCC	JPEG	JP2K	WN	GB	PN	CC
BRISQUE [13]	0.806	0.840	0.723	0.820	0.378	0.804
ILNIQE [14]	0.899	0.906	0.850	0.858	0.874	0.501
HOSA [16]	0.733	0.818	0.604	0.841	0.500	0.716
WaDIQaM [19]	0.853	0.947	**0.974**	0.979	0.882	0.923
CaHDC [22]	0.908	0.931	0.906	0.923	0.881	0.872
DBCNN [5]	0.940	0.953	0.948	0.947	0.940	0.870
HyperIQA [23]	0.934	0.960	0.927	0.915	0.931	0.874
CLIP-IQA [50]	0.921	0.923	0.902	0.906	0.938	0.853
**Ours**	**0.969**	**0.963**	0.962	**0.980**	**0.943**	**0.932**

Based on the experimental findings, it is evident that our proposed IQA method based on GCN and multi-scale features achieves SROCC values greater than 0.9 for all distortion types, this demonstrates that the pre-trained model may acquire previous knowledge to differentiate well between distortion kinds and distortion levels, and can effectively construct good distortion representation for both trained and untrained distortion types. The image’s global information is also pooled, which helps to further learn the perceptual quality.

### Ablation study

By performing ablation tests on the LIVEC and LIVE datasets, the efficacy of the modules in our suggested technique was confirmed. The SROCC findings from the trials are displayed in Table 5.

**Table 5:** Results of ablation study on the LIVEC and LIVE datasets.

Methods	LIVEC	LIVE
Resnst50	0.796	0.921
Resnet50 + SC	0.804	0.933
Resnet50 + DGR	0.819	0.950
Resnet50 + Meta	0.833	0.955
Resnet50 + DGR + SC	0.851	0.962
Resnet50 + DGR + SC+Meta	0.860	0.971
Resnet50 + DGR + SC+Meta+SPP	**0.881**	**0.979**

Firstly, for the baseline model (BL), we do not perform any pre-training operation on the model and only uses Resnet50 as the backbone to crop distorted images into specific image patches for prediction. For the BL model there is no training on distortion type and distortion level, nor is there any prior knowledge of the various distortion types. As may be seen from the table’s outcomes, the BL model achieved the lowest prediction accuracy. Next, the model was pre-trained to construct the distortion graph representation based on GCN and optimized using the distortion type discriminant module and distortion level discriminant module. The model representation was BL + DGR, which achieved a 2.89% and 3.15% improvement on SROCC, verifying the effectiveness of the graph representation for modeling distortion-related factors. The accuracy of the model prediction was then further improved by adding the adaptive fusion multi-scale features module (SC) and the meta-learning training process (Meta) to the convolutional network in turn. Finally, the SPP module is added so that the model can accept images of arbitrary scale and retain more image information and quality. The experimental results validate that the features extracted by our method are more advantageous than the CNN used alone, and therefore more in line with the perceptual characteristics of the human visual system.

We have analyzed the complexity of each module in the model, and we have listed the total parameter counts, inference time, and FLOPs of all models in Table 6. From Table 6, it can be seen that traditional methods have relatively low parameter count and computational complexity, while complex deep learning methods have relatively high parameter count and computational complexity. Our method falls between the two, achieving a good balance between accuracy and complexity.

**Table 6:** The number of parameters and computational complexity for different NR-IQA methods.

	Total parameters	Inference time	Flops
BRISQUE [13]	32.4M	2.6ms	115G
ILNIQE [14]	28.2M	2.3ms	98G
HOSA [16]	130.2M	9.8ms	457G
WaDIQaM [19]	35.6M	2.8ms	127G
CaHDC [22]	38.7M	3.0ms	138G
SFA [40]	37.6M	2.9ms	135G
DBCNN [5]	42.0M	3.3ms	152G
HyperIQA [23]	63.2M	5.7ms	211G
MetaIQ [41]	35.2M	2.7ms	127G
MetaIQA+ [42]	64.5M	4.9ms	226G
EDIIQA [43]	225.6M	16.8ms	763G
VISOR [44]	235.4M	18.1ms	814G
TempIQA [45]	312.4M	25.3ms	1251G
CLIP-IQA [50]	257.2M	19.0ms	895G
QualiCLIP [47]	259.0M	19.8ms	901G
HiRQA [48]	272.1M	16.6ms	954G
AKD-IQA [46]	321.3M	23.9ms	1124G
FsPN [49]	34.2M	9.5ms	256G
Ours	41.1M	3.2ms	143G

## Discussion

In this paper, we propose an NR-IQA method to change the convolutional operation to expand the perceptual field, adaptively construct connections between multi-scale features. In the meanwhile, robust learning is achieved by fusing the distortion information acquired from the meta-model to improve feature representation. Finally, distortion types and distortion levels are modeled as hierarchical relations based on graph representations by GCN to avoid the complexity of modeling distortion relations in convolutional networks, and the prior of image content is used to optimize the model. However, graph building networks contain a GCN operation at each level, which suffers from the Laplace smoothing problem. GCN can effectively model the relationship between distortion types and degrees, thereby obtaining distortion characteristics that are more in line with HVS. Compared to other networks, GCN construction is relatively simple, with less computational complexity, and can effectively reduce the number of model parameters and resource costs. The drawback is that most GCNs have shallow structures, making it difficult to obtain sufficient global information. However, we introduce multi-scale feature fusion to obtain more global information and improve the performance of the model. However, the model still has certain shortcomings, and there is still room for improvement in modeling the relationship between distortion types and degrees. Therefore, we will further explore to obtain richer global information using more efficient deep GNN. In addition, our adaptive fusion multi-scale features module fuses global and local information from spatial and channel dimension, but ignores the effect of semantic features of low-level networks. We will further extend the present approach in future work. Adopting a more lightweight backbone network to construct graph convolutional neural networks, such as MobileViT. Using richer multi-scale strategies to fuse global and local information to solve the problem of GCN difficulty in obtaining sufficient global information, while also enhancing the role of underlying network semantic features. Using large language models to assist in the training of graph neural networks, in order to better construct the relationship between distortion types and degrees.

## Conclusion

In this paper, an NR-IQA method based on graph convolutional networks and multi-scale features is proposed, aiming to address the problems of other IQA methods based on distortion representation and the shortcomings of existing convolutional networks. Firstly, we introduce a meta-learning training method to improve the generalization ability to unknown distortion scenes. At the same time, an adaptive fusion multi-scale feature module is introduced in the training of the meta-model, which expands the convolutional field of perception using enhanced convolutional operations and calibrates the relationship between global contexts by fusing local and global features. In this sense, the model’s performance is enhanced for later training by obtaining the fuller distortion information. Then, the spatial pyramid pooling is added after the meta-model to enable the processing of images of arbitrary size, followed by modeling the relationship between distortion type, distortion level and image content through the GCN using the designed graph representation building module. Finally, the pre-trained model experiments with the prediction of arbitrary distorted images using rich prior knowledge. Experimental results show that the proposed method achieves SROCC values greater than 0.9 for the single distortion types, and experiments on four publicly available datasets demonstrate that our method achieves competitive performance compared to methods designed specifically for synthetic or authentic distortion.
